# Comparison of statistical methods for the analysis of patient-reported outcomes (PROs), particularly the Short-Form 36 (SF-36), in randomised controlled trials (RCTs) using standardised effect size (SES): an empirical analysis

**DOI:** 10.1186/s12955-025-02373-z

**Published:** 2025-04-29

**Authors:** Yirui Qian, Stephen J. Walters, Richard M. Jacques, Laura Flight

**Affiliations:** 1 Centre for Health Economics, University of York, York, UK; 2 Division of Population Health, School of Medicine & Population Health, University of Sheffield, Sheffield, UK

**Keywords:** Standardised effect size, Statistical methods, SF-36, Patient-reported outcome, Randomised controlled trial

## Abstract

**Background:**

The Short-Form 36 (SF-36), a widely used patient-reported outcome (PRO), is a questionnaire completed by patients measuring health outcomes in clinical trials. The PRO scores can be discrete, bounded, and skewed. Various statistical methods have been suggested to analyse PRO data, but their results may not be presented on the same scale as the original score, making it difficult to interpret and compare different approaches. This study aims to unify and compare the estimates from different statistical methods for analysing PROs, particularly the SF-36, in randomised controlled trials (RCTs), using standardised effect size (SES) summary measure.

**Methods:**

SF-36 outcomes were analysed using ten statistical methods: multiple linear regression (MLR), median regression (Median), Tobit regression (Tobit), censored absolute least deviation regression (CLAD), beta-binomial regression (BB), binomial-logit-normal regression (BLN), ordered logit model (OL), ordered probit model (OP), fractional logistic regression (Frac), and beta regression (BR). Each SF-36 domain score at a specific follow-up in three clinical trials was analysed. The estimated treatment coefficients and SESs were generated, compared, and interpreted. Model fit was evaluated using the Akaike information criterion.

**Results:**

Estimated treatment coefficients from the untransformed scale-based methods (Tobit, Median, & CLAD) deviated from MLR, whereas the SESs from Tobit produced almost identical values. Transformed scale-based methods (OL, OP, BB, BLN, Frac, and BR) shared a similar pattern, except that OL generated higher absolute coefficients and BLN produced higher SESs than other methods. The SESs from Tobit, BB, OP, and Frac had better agreement against MLR than other included methods.

**Conclusions:**

The SES is a simple method to unify and compare estimates produced from various statistical methods on different scales. As these methods did not produce identical SES values, it is crucial to comprehensively understand and carefully select appropriate statistical methods, especially for analysing PROs like SF-36, to avoid drawing wrong estimates and conclusions using clinical trial data. Future research will focus on simulation analysis to compare the estimation accuracy and robustness of these methods.

**Supplementary Information:**

The online version contains supplementary material available at 10.1186/s12955-025-02373-z.

## Background

The Short-Form 36 (SF-36) is a widely used patient-reported outcome (PRO) to measure quality-of-life from patients’ perspectives in clinical trials. The SF-36 consists of eight domain scores and one health transition item using 36 items on different ordinal categorical scales. The domain scores include physical functioning (PF), role limitation – physical (RP), bodily pain (BP), general health (GH), vitality (VT), social functioning (SF), role limitation – emotional (RE), and mental health (MH) [1].

The original version of the SF-36 was initially released in 1992 [2], with its validity and reliability tested in the subsequent two years [3, 4]. Modifications of the original SF-36 (SF-36v1) include the RAND 36-item [5], a publicly available version with slightly different scoring methods of the original version [1]; the SF-36 version 2 (SF-36v2) [6], an upgraded version with improvements in wording and in different ordinal categorical scales of some items to enhance the internal reliability consistency and reduce ceiling and floor effects [7]; the Short-Form 6-Dimension (SF-6D), a popular preference-based measure producing utility scores for use in the economic evaluation [8]; and other shorter versions that use only 8 or 12 items instead of all 36 items [9].

Two types of scoring mechanisms are commonly seen to produce SF-36 domain scores: the original scoring and the norm-based scoring. The original scoring anchors each scale from 0 (worst score on all items) to 100 (best score on all items). Norm-based scoring linearly rescales the eight domain scores to achieve a mean score of 50 and a standard deviation of 10 in the reference population (i.e. the US general population) [10]. While the described methods apply to both scoring approaches, we use the original, 0 to 100, scoring here for simplicity.

The domain scores generated from the original scoring mechanism tend to be discrete, bounded, and skewed [11]. When analysing these scores with classical statistical methods (such as linear regression or *t*-test), the model assumptions (such as Normality of residuals or heteroscedasticity) are likely to be violated. An inappropriate analysis can result in unreliable estimates, with wider confidence intervals (CIs) or larger standard errors, and accordingly fail to provide accurate and robust results for decision-making [12].

Various statistical methods have been developed for the analysis of SF-36 data, or PRO data in general, and a few comparisons of these methods have been conducted [13–16]. However, some estimated treatment coefficients from different statistical methods may not be comparable in these studies. For example, the estimate of an ordered logit model is interpreted as an odds ratio after inverse log-transformation, whereas the estimate of a linear regression is a mean. The two types of estimates – odds ratio and mean – are not comparable. The standardised effect size (SES), which is calculated by dividing the group difference by the pooled standard deviation, can produce estimates with no units of measurement and therefore allows the comparison of estimates that are based on different scales [17–19]. We introduce the SES summary measure to unify the estimated treatment coefficients from different statistical methods for analysing PROs.

This study aims to apply various statistical methods for the analysis of SF-36 domain scores using data from randomised controlled trials (RCTs) and compare estimates from different methods using the SES summary measure of the treatment effect. In the rest of the paper, we first describe the included statistical methods for analysing SF-36; then produce and interpret the estimated treatment coefficients and SESs from these statistical methods; and finally present the agreement of the SESs from these statistical methods.

## Methods

A series of secondary analyses of the SF-36v2 outcome using the original scoring mechanism at a single follow-up timepoint were conducted using data from three RCTs. The scoring strategies for eight domain scores in SF-36v2 are presented in Table 1.

**Table 1:** Scoring strategies for eight domains in SF-36 version 2

SF-36 Domain	Abbr	No. items	No. levels	Raw score range	No. possible values after recoding
Physical functioning	**PF**	10	21	10 to 30	21
Role limitation – physical	**RP**	4	17	4 to 20	17
Bodily pain	**BP**	2	27	2 to 11	10
General health	**GH**	5	39	5 to 25	21
Vitality	**VT**	4	17	4 to 20	17
Social functioning	**SF**	2	9	2 to 10	9
Role limitation – emotional	**RE**	3	13	3 to 15	13
Mental health	**MH**	5	21	5 to 25	21

*Abbr *abbreviation, *SF-36 *Short-Form 36

### Description of statistical methods

Ten statistical methods included for comparison were multiple linear regression (MLR), median regression (Median), Tobit regression (Tobit), censored absolute least deviation regression (CLAD), beta-binomial regression (BB), binomial-logit-normal regression (BLN), ordered logit model (OL), ordered probit model (OP), fractional logistic regression (Frac) and beta regression (BR). The selection of these ten statistical methods were based on their widespread use, ability to accommodate different distributions for the outcome, applicability to various scenarios, and recommendations from previous studies [14, 20], originating from our previous review that summarised a list of statistical methods for PRO analysis [21]. These methods are described under the generalized linear model (GLM) framework [22] and presented in Table 2. The SF-36 domain score at a specific post-randomisation timepoint in each trial was analysed by these ten statistical methods, comparing the treatment groups and adjusting for the corresponding baseline scores, as the treatment effect is regarded as the main outcome of clinical trials, and a PRO score is likely to correlate with its baseline score [20, 23].

**Table 2:** Summary of ten statistical methods for the analysis of SF-36 dimension scores under the GLM framework

**Statistical methods**	**Distribution of the outcome/dependent variable (** ${\varvec{Y}}$**)**	**Link function** ${\varvec{g}}(\bullet )$	**Model assumption**	**Recoding of PRO needed**	**Interpretation**	**Estimation method**	**Stata command**
*Classical model*							
MLR	Continuous	Identity $g\left(\mu \right)=\mu ={\varvec{X}}{\varvec{\beta}}$	Normality (of residuals); homoscedasticity; linearity; independence of outcomes	No	Mean	OLS or MLE	regress
Median	Continuous	${g\left({Q}_{Y|X}\left(median\right)\right)=Q}_{Y|X}\left(median\right)={\varvec{X}}{{\varvec{\beta}}}_{median}$	Linearity; independence of outcomes	No	Median	LAD	qreg
*Censored regression*							
Tobit	Observed$Y$: Continuous and censored; Latent${Y}^{*}$: Continuous	Identity $g\left({\mu }^{*}\right)={\mu }^{*}={\varvec{X}}{\varvec{\beta}}$	Normality (of residuals); homoscedasticity; linearity; independence of outcomes	No	Latent Mean	MLE	tobit
CLAD	Observed$Y$: Continuous and censored; Latent${Y}^{*}$: Continuous	${g\left({Q}_{{Y}^{*}|X}\left(median\right)\right)=Q}_{{Y}^{*}|X}\left(median\right)={\varvec{X}}{{\varvec{\beta}}}_{median}$	Linearity; independence of outcomes	No	Latent Median	CLAD	clad
*Ordinal regression*							
OL	Ordinal	Logit $g({\theta }_{il})=\text{ln}(\frac{{\theta }_{il}}{1-{\theta }_{il}})$	Proportional-odds; linearity; independence of outcomes	Yes (to [0,$k$−1] ⊆ ℕ)	Odds ratio	MLE	ologit
OP	Ordinal	Probit ${g\left({\theta }_{il}\right)=\Phi }^{-1}\left({\theta }_{il}\right)$	Proportional odds; linearity; independence of outcomes	Yes (to [0,$k$−1] ⊆ ℕ)	Probability	MLE	oprobit
*Binomial regression*							
BB	Beta-binomial i.e.${Y}_{i}\sim Bin(k,{\theta }_{i})$ ${\theta }_{i}\sim Beta(\alpha ,\gamma )$	Logit $g({\theta }_{i})=\text{ln}(\frac{{\theta }_{i}}{1-{\theta }_{i}})$	Linearity; independence of outcomes; beta distribution of probability of success	Yes (to [0,$k$−1] ⊆ ℕ)	Odds ratio	MLE	betabin
BLN	Binomial-logit-Normal i.e.${Y}_{i}\sim Bin\left(k,{\theta }_{i}\right)$ ${\theta }_{i}\sim LN(\text{0,1})$	Logit $g({\theta }_{i})=\text{ln}(\frac{{\theta }_{i}}{1-{\theta }_{i}})$	Linearity; independence of outcomes; logit-Normal distribution of probability of success	Yes (to [0,$k$−1] ⊆ ℕ)	Odds ratio	MLE	glm…link(logit) family(binomial N)
*Fractional regression*							
Frac (logit link)	Recoded$Y{\prime}$: continuous and bounded in [0, 1]	Logit $g({\mu}_{Y^{\prime}})=\text{ln}(\frac{{\mu }_{Y^{\prime}}}{1-{\mu}_{Y^{\prime}}})$	Linearity; independence of outcomes	Yes (to [0, 1] scale)	Odds ratio	Quasi-likelihood estimation	fracreg logit
BR (logit link)	Recoded$Y{\prime}{\prime}$: continuous and bounded in (0, 1) $Y{\prime}{\prime}\sim Beta(\mu \varphi , \left(1-\mu \right)\varphi )$	Logit $g({\mu}_{Y^{\prime\prime}})=\text{ln}(\frac{{\mu}_{Y^{\prime}}}{1-{\mu }_{Y^{\prime\prime}}})$	Linearity; independence of outcomes	Yes (to (0, 1) scale)	Odds ratio	MLE	betareg

*BB *Beta-binomial regression, *BLN *binomial-logit-Normal regression, *CI *confidence interval, *CLAD *censored least absolute deviations, *Frac *fractional logistic regression, *GLM *generalized linear model, *LAD *least absolute deviations, *Median *median regression, *MCAR *missing completely at random, *MCDA *multi-criteria decision analysis, *MLE *maximum likelihood estimation, *MLR *multiple linear regression, *SF-36 *Short-Form 36, *Tobit *Tobit regression, *OL *ordered logit model, *OLS *ordinary least squared, *OP *ordered probit model, *PRO *patient-reported outcomes.$k$, represents the number of possible categorical values in a domain;$\mu$denotes the mean;${\mu }^{*}$denotes the latent mean; ℕ, denotes the non-zero positive natural numbers i.e. 1,2,3…$k$−1;$\varphi$is the precision parameter for beta distribution;$\Phi$stands for the standard Normal cumulative distribution function.$\theta$, denotes the probability of success or the cumulative response probabilities i.e.${\theta }_{il}=P(Y\le l)$the probability of a response in category$l$or below for OL, and${\theta }_{i}=P(Y=l)$the probability of a response in category$l$for BB, BLN, and BR. Note that clad and betabin are user-developed packages in Stata, and therefore installation of the corresponding package is required to run these two commands

MLR is the most commonly used method for the analysis of PROs with some appealing features: it requires no transformation of the response variable, it produces point estimates that are based on the untransformed scale of measurement and are easy to interpret, and it is a robust method when faced with the violation of model assumptions [24]. Tobit is known to be consistent and efficient under the Normality assumption and homoscedasticity [25, 26]. When these model assumptions are violated, CLAD can be used as a substitute for Tobit [27]. It is worth noting that the use of Tobit or CLAD relies on the premise that a PRO score exceeding the low or/and high boundaries is possible and meaningful [28]. Two ordinal regression methods, OL and OP, assume proportional odds. Compared to OL, OP may be less preferable to use, since the estimated treatment coefficient from an OP cannot be explained in odds ratio as the OL does. BB and BLN are built upon binomial regression, with their probability parameter being a random variable and following beta distribution and logit-Normal distribution respectively [13, 14, 29]. They both can account for the ordinal and discrete feature of the PRO scores without the requirement for distributional assumptions. Fractional regression is suitable to analyse bounded data falling between 0 and 1 [30], such that recoding the SF-36 domain score that are on a [0, 100] scale is needed to apply fractional regression. Frac can account for scores at boundaries of 0 and 100, whereas BR cannot, and it therefore requires the ‘squeezing’ of the domain scores [16].

Model assumptions were tested where necessary, including the Normality of residuals and the homoscedasticity assumptions for MLR and Tobit, and proportional odds assumptions for ordinal regression. To run statistical methods that require outcome variables on transformed scales, different recoding techniques were used to transform the SF-36 domain scores to appropriate forms [31–33]. Technical details of these methods and the recoding strategies for the SF-36 domain scores are summarised in the Supplementary Material.

### Estimated treatment coefficient and standardised effect size

An estimand is a well-defined and explicit description of precisely what treatment effect is to be estimated in an RCT [34]. It consists of five connected elements [35]: the treatments to be compared, the target population, the outcome or endpoint, intercurrent event handling, and a population-level summary measures of how outcomes between the different randomised groups will be compared. Common population-level summary measures include the difference in means, risk ratios and odds ratios.

If the treatment coefficient is chosen as the summary measure to compare outcomes between the different randomised groups, then it may not make sense to compare the ten estimators (i.e. the statistical methods) and their associated estimates as they are different estimands. However, some of the methods, such as MLR, Tobit, CLAD, and Median produce estimates that have similar population summary measures that look at differences in location or central tendency e.g. differences in means, or medians. Therefore, in these models it may be sensible to compare the treatment coefficient estimates of difference in means or medians between two treatment groups produced by these models as they are similar estimands. Again, some of the methods, such as OL, BB, BLN, Frac, and BR, also have similar population summary measures that estimate the (log) odds ratio for the treatment effect. Therefore, in these models it may be sensible to compare the estimates of (log) odd ratios between two treatment groups produced by these models, as they are similar estimands.

The estimates of MLR, Tobit, CLAD and Median are generated using SF-36 based on the untransformed scale. As the logit link is used for OL, BB, BLN, BR, and Frac, their estimated treatment coefficients can be interpreted as odds ratio through the exponential transformation, where $coef\left(TE\right)$ denotes the estimated values for the treatment effect parameter.

$${Odds Ratio}_{TE}=\text{exp}(coef\left(TE\right))$$

A special case of these methods is the OP, which uses a probit link. The probit is the inverse of the cumulative standard Normal distribution that is denoted by $\Phi$.The estimates of an OP cannot be transformed to odds ratios as other methods do, but it can be interpreted as the probability or the effect size in the response.

Under the estimand framework, we can compare the estimates of the treatment difference between the treatment groups from the different statistical methods, using the SES as the population level summary measure, as the other four attributes for the estimand (the treatment, the target population, the outcome, and intercurrent event handling) are the same regardless of the statistical methods used. The ten statistical methods that we compared in this study all fit under the GLM framework, and their Z-statistics are calculated using the same formula, i.e. the point estimate, of the treatment effect parameter, divided by its standard error. Therefore, in these circumstances, the estimand, and its population summary measure, the SES, is the same, but the estimators (e.g. the ten statistical models) will be different and may produce different estimates that can be compared and contrasted.

After producing estimated treatment coefficients from the different statistical methods, their scale-invariant SES and its associated standard error were calculated using the following formula [18, 36]:

$$SES=Z \times \sqrt{\frac{1}{{n}_{1}}+\frac{1}{{n}_{2}}}=\frac{coef(TE)}{SE(TE)}\times \sqrt{\frac{1}{{n}_{1}}+\frac{1}{{n}_{2}}}$$

$$SE\left(SES\right)=\sqrt{\frac{{{n}_{1}+n}_{2}}{{n}_{1}{n}_{2}}+\frac{{SES}^{2}}{2({n}_{1}+{n}_{2})}}$$

where Z stands for the Z-statistics; *TE* stands for the treatment effect; $coef(TE)$ stands for the estimated values for the treatment effect parameter; $SE(TE$) stands for the standard error of the treatment effect estimates; $SE\left(SES\right)$ represents the standard error of the SES; and ${n}_{1}$ and ${n}_{2}$ represents the sample size in each treatment group respectively.

The Akaike information criterion (AIC) [37] values were produced when fitting different statistical methods to compare the model fit, using the following equation:

$$AIC=2c-2 \text{ln}(\widehat{L})$$

where $\widehat{L}$ is the maximum likelihood for the model, and $c$ is the number of estimated parameters.

A lower AIC value represents a better model fit. Note that the value $c$ for different statistical methods with the same set of independent variables can be different, and the AIC values cannot be calculated for CLAD and Median because they both are quantile regression methods which use (censored) least absolute deviation for estimation rather than maximum likelihood.

The characteristics of the three trials included in this study were summarised in a table by the basic trial information on the study population, primary outcome, follow-up timepoints, samples size at the baseline, and number of patients analysed. Scatter plots were generated to compare the estimated treatment coefficients from different methods, using consistent markers for each method and consistent colours for each trial. Estimated treatment coefficients from the MLR and BB were used as the reference benchmark for statistical methods on the untransformed and transformed scales respectively, since MLR is the most commonly used methods for analysing PROs [38], and BB was reported to render satisfactory results in various situations for PRO analysis [14]. The SESs from different methods were displayed in scatter plots, using MLR as the reference benchmark for all included methods regardless of their scales, as the SES is believed comparable among statistical methods on different scales under the estimand framework. Effect size plots were graphed for the SESs with its associated CIs of estimated SES from ten statistical methods, together with two horizontal lines representing the clinical and statistical significance. The change of AICs against a number of possible categorical values (i.e. levels) of SF-36 domain scores was graphed using scatter plots.

We used the statistical package Stata/MP 17.0 for statistical analysis and data visualisation. Stata codes for applying the recoding techniques and conducting regression analyses are available in the Supplementary Material.

## Results

### Description of datasets

The three RCTs included in this study are Chronic Obstructive Pulmonary Disease (COPD) [39], Lifestyle Matters (LM) [40] and Putting Life IN Years (PLINY) [41] (Table 3). The follow-up timepoints for analysis were chosen as 2 months (acute phase endpoint) for COPD, and 6 months (primary endpoint) for both LM and PLINY. Each statistical method produced 24 estimates of treatment effect for eight SF-36 domain scores across the three RCTs. A total number of 684 patients were randomised in the three trials combined, and 492 patients that have SF-36 outcome data at follow-up were analysed.

**Table 3:** Characteristics of the three RCTs that used SF-36 domain scores as clinical outcomes

Trial name	Trial population	Primary outcome	Follow-up timepoints (months)	Sample size at baseline			Max sample size analysed (PRO)			Ref
				**Total**	**Control**	**Treat**	**Total**	**Control**	**Treat**	
**COPD**	Patients with chronic obstructive pulmonary disease	Difference in improvement in endurance shuttle walking test (ESWT) during 18 months follow-up	**2**, 6, 12, 18	239	129	110	174	93	81	[38]
**LM**^a^	Independently living older people (aged 65 or more)	SF-36v2 MH at 6 months follow-up	**6**, 24	288	143	145	262	126	136	[39]
**PLINY**^a^	Independently living older people (aged 75 or more)	SF-36v2 MH at 6 months follow-up	**6**	157	79	78	56	30	26	[40]
***Total***				*684*	*351*	*333*	*492*	*249*	*243*	

^a^Trials using SF-36 domains as primary outcomes. PLINY and LM used SF-36v2 MH at 6 months as primary clinical outcomes

*MH mental health, PRO *patient-reported outcome, *SF-36v2* Short-Form 36 version 2

In general, skewed distributions of residuals and heteroscedasticity are shown in SF-36 domain scores after the post estimation of MLR and Tobit, and 20.8% of the model outcomes using ordinal regression violated the proportional odds assumption. A summary of post-estimation plots for MLR and Tobit are available in the Supplementary Material (Figure S1-S4).

### Estimated treatment coefficients and interpretation

**Fig. 1:**
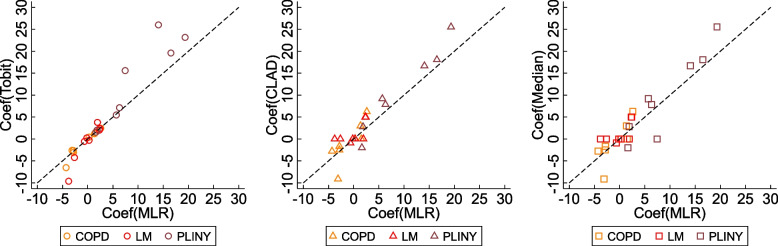
Estimated treatment coefficients from untransformed scale-based statistical methods against MLR. Coef, treatment coefficient; CLAD, censored absolute least deviation regression; Median, median regression; MLR, multiple linear regression; Tobit, Tobit regression

**Fig. 2:**
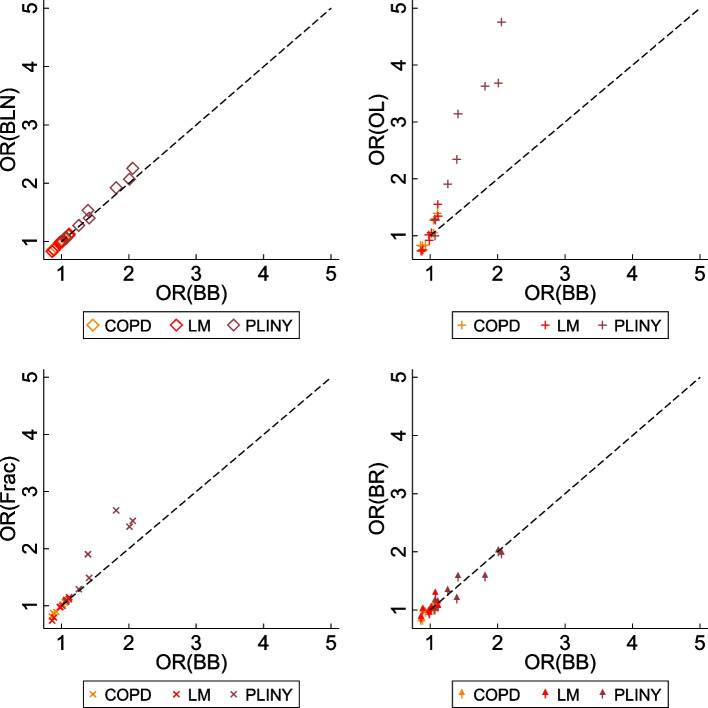
Estimated ORs from transformed scale-based statistical methods against BB. BB, beta-binomial regression; BLN, binomial-logit-normal regression; BR, beta regression; Frac, fractional logistic regression; OL, ordered logit model; OR, odds ratio

Figure 1 and Fig. 2 show the scatter plots of estimated treatment coefficients from the untransformed scale-based methods against MLR and the transformed scale-based methods against BB respectively. Estimates from statistical methods on the untransformed scale (i.e. Tobit, CLAD, and Median) deviated from MLR. Especially when the magnitude of the estimated treatment effects was large, they tended to produce higher estimates than MLR. CLAD and Median shared a similar pattern when the estimates were small, i.e. they tended to produce estimates scattering at zero. Estimates from methods on the transformed scales were presented using odds ratios, except for OP. The odds ratios estimated from BLN and Frac are shown similar to BB. The OL produced higher absolute estimates than other methods, and the exponentiation procedure to generate odds ratios magnified this trend. This trend is obvious in PLINY that presented averagely higher treatment estimates than other trials.

An example of how to interpret the estimated treatment coefficients from the included ten statistical methods is presented in Table 4, based on the SF-36 MH score at 6-month follow-up in the LM trial.

**Table 4:** Interpretation of the treatment coefficient from the ten statistical methods using the SF-36 MH score at 6-month follow-up in the LM trial as an example

(a) Statistical methods that used the untransformed scale			
**Statistical methods**	**Coefficient**	**Interpretation**	
MLR	2.31	The mean of the MH score at 6 months for the treatment group is 2.31 points higher than the mean for the control group, after adjusting for baseline MH score	
Tobit	2.02	The mean of the *uncensored* MH score at 6 months for the treatment group is 2.02 points higher than the mean for the control group, after adjusting for baseline MH score	
Median	5.00	The median of the MH score at 6 months for the treatment group is 5.00 points higher than the median for the control group, after adjusting for baseline MH score	
CLAD	5.00	The median of the *uncensored* MH score at 6 months for the treatment group is 5.00 points higher than the median for the control group, after adjusting for baseline MH score	
(b) Statistical methods that used the transformed scales			
**Statistical methods**	**Coefficient**	**Odds**	**Interpretation**
BB	0.11	1.12	The odds of the MH score being in a given category at 6 months in the treatment group is 1.12 times that of the odds for the control group, after adjusting for baseline MH score
BLN	0.12	1.13	The odds of the MH score being in a given category at 6 months in the treatment group is 1.13 times that of the odds for the control group, after adjusting for baseline MH score
OL	0.29	1.34	The odds of the MH score being in a given category **or less** at 6 months in the treatment group is 1.34 times that of the odds for the control group, after adjusting for baseline MH score
OP	0.16	NA	*(Marginal effects need to be calculated to generate the probability)* The probability of scoring 90.0 at 6 months is 21.2% for the treatment group and 20.1% for the usual care group, after adjusting for baseline MH score
Frac	0.14	1.15	The odds of the MH score being in a given category at 6 months in the treatment group is 1.15 times that of the odds for the control group, after adjusting for baseline MH score
BR	0.04	1.05	The odds of the MH score being in a given category at 6 months in the treatment group is 1.05 times that of the odds for the control group, after adjusting for baseline MH score

*BB *beta-binomial regression, *BLN *binomial-logit-normal regression, *BR *beta regression, *CLAD *censored absolute least deviation regression, *Frac *fractional logistic regression, *OL *ordered logit model, *OP *ordered probit, *Median *median regression, MH mental health, *MLR *multiple linear regression, *NA *not applicable, *SF-36 *Short-Form 36, *Tobit *Tobit regression

### Comparison of estimated standardised effect sizes

Overall, the estimated SESs in our datasets were small (i.e. absolute values less than 0.2), except for the SESs of some domains in PLINY (i.e. absolute values between 0.5 and 1.4). CLAD failed to converge on one occasion. When estimating the treatment coefficient of the same response using different methods, SESs with different directions were produced from ten statistical methods. However, there was no case where these statistical methods produced statistically significant estimates with different directions. For SESs with the same direction, different statistical significance and magnitudes of effect size are observed in these analyses.

**Fig. 3:**
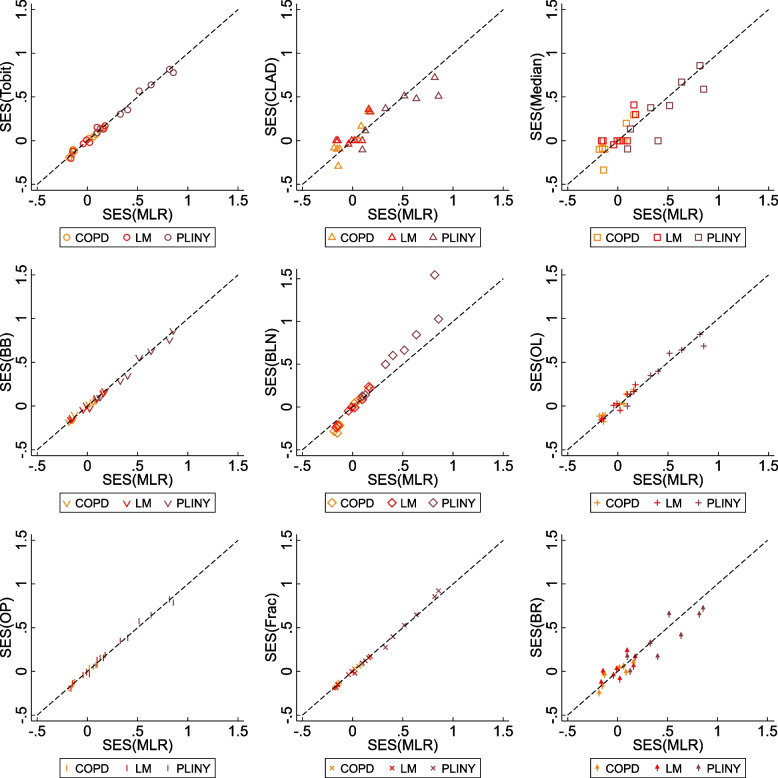
Estimated SES from different statistical methods against MLR. The x-axis of the scatter plots is the SES estimated by MLR, and the y-axis is the SES estimated by other statistical methods. The black dash line represents the method that produces the same SES as MLR. BB, beta-binomial regression; BLN, binomial-logit-normal regression; BR, beta regression; CLAD, censored least absolute deviation regression; Frac, fractional logistic regression; Median, median regression; MLR, multiple linear regression; OL, ordered logit model, OP, ordered probit model; Tobit, Tobit regression; SES, standardised effect size

Figure 3 presents the SES estimated from ten different statistical methods against MLR. For statistical methods that used the untransformed scale of measurement, Tobit has almost identical pattern against MLR, whereas CLAD and Median are more scattered. For methods that used transformed scales, the OL that produced higher estimated coefficients (or odds ratio) showed similar SESs as other methods after standardisation. Conversely, although BLN produced similar estimated coefficients (or odds ratios) as BB, the SESs from BLN was larger than other methods after standardisation. It also shows that the Tobit, BB, OP, and Frac had stronger agreement with MLR across the three included trials, i.e. the difference between each of these four methods against MLR is associated with less bias and narrower 95% CIs than rest of the methods.

**Fig. 4:**
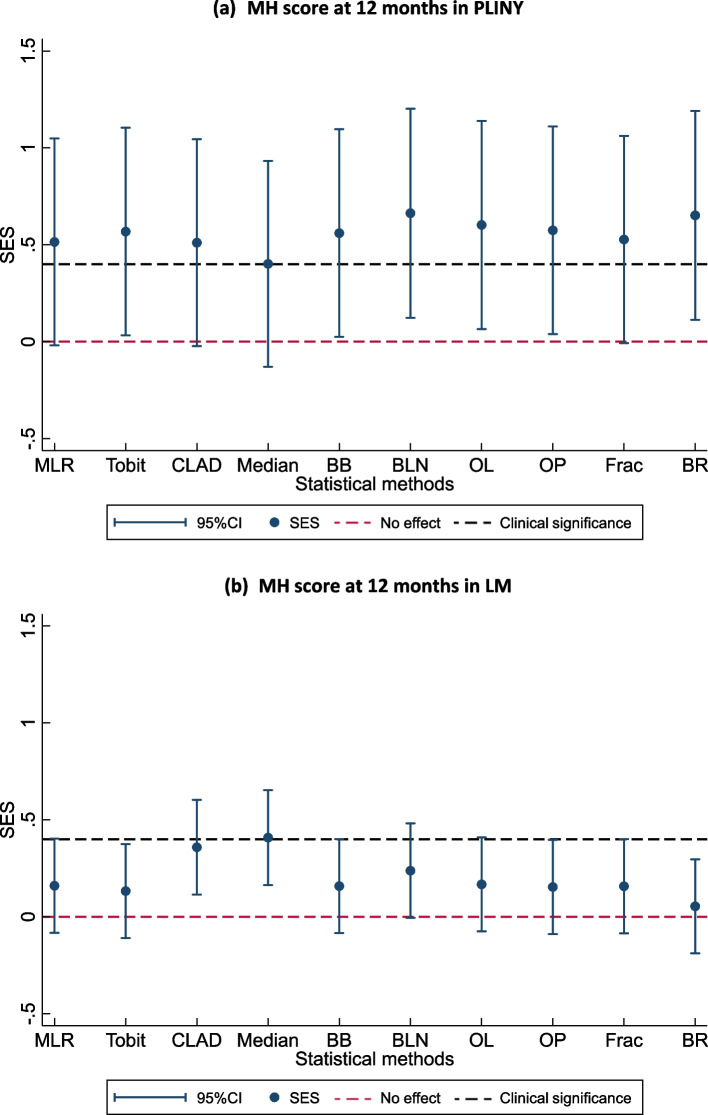
SES with associated 95% CIs from ten statistical methods BB, beta-binomial regression; BLN, binomial-logit-normal regression; BR, beta regression; CLAD, censored least absolute deviation regression; Frac, fractional logistic regression; Median, median regression; MLR, multiple linear regression; OL, ordered logit model, OP, ordered probit model; Tobit, Tobit regression; SE, standard error; SES, standardised effect size. The bars on two sides of the vertical line for each method represents the 95% confidence intervals (CIs) for SES, which are calculated using $SES\pm 1.96\times SE(SES)$, where $SE\left(SES\right)$ represents the standard error of the SES

Figure 4 shows the SES with its associated 95% CIs of treatment estimates from different statistical methods in two trials (PLINY and LM) that used SF-36 MH score at 6-month follow-up as primary outcome. Two horizontal lines are drawn in the plot, representing the SES having no effect or no difference between two treatment groups (i.e. y = 0) and clinical significance (i.e. y = minimal clinical important difference/standard deviation = 0.4 in both trials). When analysing the treatment effect of the same outcome, the use of different statistical methods may draw different results in terms of statistical significance and/or clinical significance. For example, SES from Median is statistically significant in LM, whereas this is not the case for MLR. In PLINY, most methods showed statistically significant estimates except for MLR, CLAD and Median. Effect size plots for other SF-36 domain scores used as secondary outcomes in these three trials are available in Supplementary Material Figure S5.

**Fig. 5:**
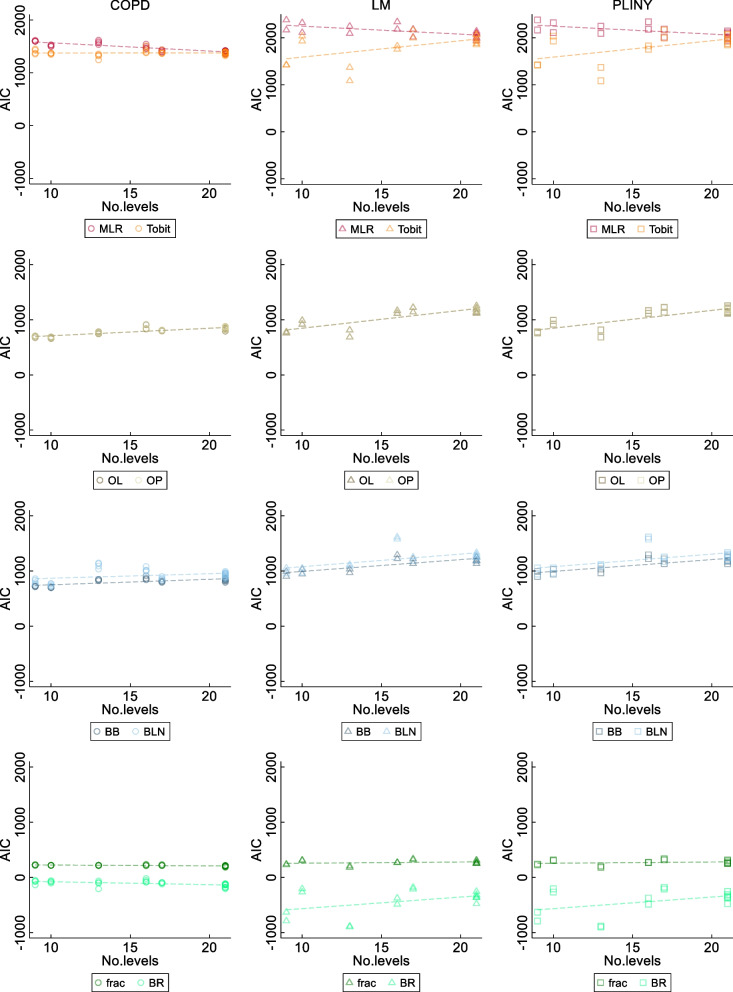
Scatter plot of AIC for different statistical methods against the number of possible observable values of SF-36 domains in three RCT datasets. Note that median and CLAD regression do not have AIC scores, and thus are not compared in this figure. These methods are classified according to their distributional assumptions on SF-36 domain scores. AIC, Akaike information criterion; MLR, multiple linear regression; Tobit, Tobit regression; OL, ordered logit model, OP, ordered probit model, BB, beta-binomial regression; BLN, binomial-logit-normal regression; frac, fractional logistic regression; BR, beta regression

Figure 5 shows how AIC changed against a different number of possible categorical values (i.e. levels) of domain scores when applying the ten statistical methods in three RCTs. As the comparison of AICs require the methods to model the same response variable [37], these methods were categorised into four groups according to their distributional assumptions and recoding techniques on SF-36 domain scores. When fitting higher levels of SF-36 domain scores, the AICs of Tobit, ordinal, and binomial regression became larger, representing a poorer fit, whereas the AICs for the MLR became smaller, representing a better fit. The scatter plot shows that AIC values for BR were not sensitive to the change of possible categorical values of domain scores.

## Discussion

This study applied ten statistical methods for the analysis of PROs to three RCTs using SF-36. A total of 240 estimates of treatment coefficients for SF-36 eight domain scores at a single follow-up timepoint were calculated across three RCTs using MLR, Median, Tobit, CLAD, BB, BLN, OL, OP, Frac, and BR. Their method theory under the GLM framework and interpretation for estimates generated from these methods were explained and presented. The SES was applied to compare the magnitude of estimated treatment coefficients from these methods based on different scales. The AIC statistics were calculated to present the change of model fit against a different number of possible values in SF-36 domain scores.

Our empirical analysis shows that SESs estimated from different methods are generally consistent, using MLR as the reference benchmark, although the estimated treatment coefficients by different methods vary. For example, the magnitude of estimated treatment coefficients by Tobit is larger than MLR, but the estimated SESs by Tobit and MLR are almost identical. This may result from the fact that the Tobit accounts for the censoring and scatters the observed variable beyond the boundaries, the latent variable of which is assumed to have a wider scale than the observed variable that is used for estimation by MLR. However, adjusting the standard deviation of treatment estimates offsets the large magnitude of the estimated coefficients, and thus results in the agreement in SES between Tobit and MLR. The CLAD, the other method applied in this study that can account for boundedness of the outcome, was found to be inefficient compared to other methods, as it took longer to run in Stata and failed to converge on one occasion. For statistical methods that used the untransformed scale, quantile regressions (Median and CLAD) show more variation. This may be because they adopt a different estimation method (i.e. least absolute deviation) in comparison to those that use maximum likelihood estimation. As was found in another study comparing Tobit, Median, and CLAD using Health Utility Index (HUI) [27], Median and CLAD tend to produce estimates with similar patterns and their estimates tend to be shrunk to zero compared to MLR and Tobit.

It is possible for different combinations of treatment coefficient and standard error estimates to produce the same effect size on the standardised scale [17]. An example for the transformed scale-based methods is the OL, which generally produced higher estimates than other methods, resulting in higher estimated odds ratios. However, the standard errors estimated from OL were also higher than other methods, offsetting the high values of estimates when calculating the SES values. Conversely, BLN produced slightly higher SES estimates, whereas the treatment coefficients from it were similar to other methods. This may result from the fact that BLN assumes a Normal distribution for random effects, but this assumption may not be valid for some SF-36 domain scores [14].

In comparison to Frac, the SES from BR biases slightly from the reference benchmark, MLR. This may be caused by the required ‘squeezing’ procedure in BR, which can reduce the estimation precision [33]. Fractional regression methods are more suitable for the analysis of health utility scores, compared to other included methods. But it is noteworthy that, in scenarios where health utilities index scatter on slightly different scales, e.g. SF-6D scattering between 0.291 and 1 for the United Kingdom value set [8], application of these two fractional regression methods may not be straightforward [33, 42].

Generally, when increasing the number of possible categorical values, the AIC for statistical methods with logit or probit link increased; it decreased, however, for MLR. Interestingly, Tobit, an extension of MLR designed to adapt for censored outcomes, generated lower AIC values (i.e. better model fit) when analysing outcomes with a small number of possible categorical values. This shows an adverse trend compared to MLR and requires further investigation.

To achieve the aim of comparing the estimates by different statistical methods that are based on different scales, we have adapted the SES as the population summary measure of the treatment effect in the estimand framework, which is not as frequently seen as other population summary measures of the treatment effect such as means, risks, or odds ratios [43]. In practice, the concept of SES has been applied in various scenarios in trials using PROs and their related studies. This includes summary studies such as meta-analysis in literature reviews that compare PROs that are based on different scales, sample size calculation in trial designs that use PRO as primary outcomes, and trials with PROs that used the effect size as the measurement of treatment effectiveness [44–48].

For linear models, the effect size is a ratio of estimated coefficient over standard deviation of the estimate. The standardisation procedure is completed by the Z statistics, adjusting for sample size. Therefore, when estimating the same treatment effect using different methods, the SES assesses the statistical power of these methods for a given sample size. For instance, if the data is ordinal and the model assumption of OL is satisfied, the OL is likely to have higher power than other statistical methods, and thus be the most appropriate method for analysing the data. In theory, the most appropriate method is more likely to capture the ‘truth’ than other methods. However, as the domain scores of SF-36 have different categories and distribution patterns, it is therefore difficult to assign them to a certain type of distribution and to decide what statistical methods to use for analysis.

Violation of model assumptions may suggest that an inappropriate statistical method is used, which may lead to inaccurate standard errors, misleading statistical significance, unreliable treatment effect estimates, and even alter the results of decision-making [49]. For example, patients may fail to receive an effective treatment because this treatment is falsely shown not to be clinically effective based on inaccurate estimates; vice-versa, patients can receive a treatment which may potentially harm their health when unreliable evidence supports the use of this treatment. Therefore, applying appropriate statistical methods for the analysis of PROs in trials is crucial to reduce biases of estimates, to accurately evaluate clinical effectiveness and to support healthcare decision-making.

It is important to understand different statistical methods before selecting and applying them [50]. Our comparison of SES estimates with its associated 95% CIs suggested that the choice of statistical methods for data analysis might result in different conclusions drawn from the hypothesis tests in terms of the clinical and statistical significance. Other factors that need to be considered for the selection of a method for the analysis of PROs include the aim of the study, the statistical features of the PRO scores, the complexity in the interpretation of a model coefficient, the computing time to run a method, and the software programs and packages availability to apply a method [20, 51].

This study has the following limitations: First, we included three trials that focused on different disease areas and populations, which can be seen as a source of heterogeneity. However, this study does not intend to compare the size of treatment estimates across different trials but to compare whether different statistical methods can produce similar estimates using the SES approach. Therefore, the results of this study should not be influenced by the magnitude of effect sizes and heterogeneity in trials.

Second, this study focused on domain scores in SF-36v2, and extrapolation to other versions of SF-36 and other types of PROs may require further validation. However, the SF-36 is a widely used generic PRO and it may be plausible to extrapolate the results to other PROs that share similar data features (discrete, bounded, and skewed) of SF-36, such as the Beck Depression Inventory (BDI), Hospital Anxiety and Depression Scale (HADS), European Organization for the Research and Treatment of Cancer Quality of Life Questionnaire (EORTC QLQ-C30), and potentially preference-based PROs such as SF-6D, and EuroQol-5 Dimensions (EQ-5D).

Third, as the aim of this study is to compare different statistical methods but not to identify the best model, we kept the analysis models in simple and similar forms, i.e. we only adjusted for the treatment group and the corresponding baseline score. Other potential effects such as time and clustering were not considered. However, the majority of the statistical methods included in this study are under the GLM framework and can be extended for longitudinal analysis by using generalized linear mixed models with coefficients estimated by maximum likelihood estimation or GLM with coefficient estimated by generalized estimating equations [43].

Fourth, our empirical analysis is based on the real case data such that the ‘truth’ of the treatment effect is unknown [52, 53], so we are not able to evaluate which statistical methods have less bias than other methods using results from this empirical analysis. Using empirical data from three RCTs in this study can show how robust the methods could be when applied to real case data. However, it still needs further investigation on how close the estimates produced by these methods are to the predefined ‘truth’, and which method remains robust when analysing different domain scores of the SF-36 and when model assumptions are violated. Our future research will focus on computational simulation to evaluate these statistical methods in terms of the accuracy of estimations and model robustness in different scenarios.

## Conclusion

In this study, estimates of treatment effect from ten statistical methods are generated and compared for the analysis of PROs in RCTs. It shows the possibility to use the SES summary measure to unify and therefore allow the comparison of estimates from various statistical methods on different scales. It is worth highlighting that these methods did not produce identical SES values, indicating the selection of inappropriate statistical methods may result in wrong estimates and conclusions when analysing clinical trial data. It is, therefore, crucial to comprehensively understand and carefully select appropriate statistical methods, especially for analysing SF-36 type data that tend to be discrete, bounded, and skewed. Future research involves using simulation methods to compare the accuracy and robustness of these methods to analyse PROs in various scenarios.

## Supplementary Information

Additional file 1.

## Data Availability

The datasets generated and/or analysed during the current study are not publicly available but may be available from the corresponding author on reasonable request.

## Abbreviations

BB: beta-binomial regression
BDI: Beck Depression Inventory
BLN: binomial-logit-normal regression
BP: bodily pain
BR: beta regression
CLAD: censored absolute least deviation regression
Coef: treatment coefficient
EORTC QLQ-C30: European Organization for the Research and Treatment of Cancer Quality of Life Questionnaire
EQ-5D: EuroQol-5 Dimensions
Frac: fractional logistic regression
GH: general health
GLM: generalized linear model
HADS: Hospital Anxiety and Depression Scale
HUI: Health Utility Index
LAD: least absolute deviation
Median: median regression
MH: mental health
MLE: maximum likelihood estimation
MLR: multiple linear regression
OL: ordered logit model
OLS: ordinary least squared
OP: ordered probit model
OR: odds ratio
PF: physical functioning
PRO: patient-reported outcome
RCT: randomised controlled trials
RE: role limitation – emotional
RP: role limitation – physical
SES: standardised effect size
SF: social functioning
SF-36: Short-Form 36
SF-6D: Short-Form 6-Dimension
Tobit: Tobit regression
VT: vitality
