# Supplementary material for: Comparison of statistical methods for the analysis of patient-reported outcomes (PROs), particularly the Short-Form 36 (SF-36), in randomised controlled trials (RCTs) using standardised effect size (SES): an empirical analysis
Source: Health Qual Life Outcomes. 2025 Apr 29;23:45. doi: 10.1186/s12955-025-02373-z (PMC12042522; doi:10.1186/s12955-025-02373-z)
Supplement: Supplementary file 1 — Additional file 1. [file 12955_2025_2373_MOESM1_ESM.docx]

**Supplementary Material for Comparison of statistical methods for the analysis of patient-reported outcomes (PROs), particularly the Short-Form 36 (SF-36), in randomised controlled trials (RCTs) using standardised effect size (SES): an empirical analysis**

**Technical details on ten statistical methods and recoding strategies of SF-36 domain scores**

Key components of a generalized linear model (GLM) are:

1. An outcome or dependent variable, denoted by $Y$, whose distribution with parameter, denoted by $\pi$, is assumed to follow a particular distribution from the exponential family;
2. A set of independent variables, denoted by $\boldsymbol{X}$, that provide a linear predictor, denoted by $\boldsymbol{X\beta}$, for $Y$; and
3. A link function $g(\cdot)$ that connects the parameter ($\pi$) and the linear predictor ($\boldsymbol{X\beta}$), i.e. $g\left( \pi\right)=\boldsymbol{X\beta}$.

In the case of analysing SF-36 scores, each domain at a specific follow-up timepoint ($Y$) is analysed separately, with the independent variables ($\boldsymbol{X}$) being the treatment group and the corresponding baseline score, to detect the treatment effect on SF-36 domain scores. The parameter ($\pi$) is defined as the mean value of PRO score ($\mu$) for methods with the identity link, as the median of PRO score, denoted by $Q_{Y|X}\left( median \right)$, for Median and CLAD, and as the mean value of the probability of success, denoted by $\theta$ for methods with logit or probit link. The probability of success represents the probability that the PRO score, $Y$, is less than or equal to particular score or category $l$, i.e. $P(Y\leq l)$ for the ordinal regression methods, or represents the probability of the PRO score being the discrete value or category $l$*,* i.e. $P(Y=l)$ for the binomial and fractional regression methods*.*

In multiple linear regression (MLR), the relationship between the mean of each SF-36 domain score and the linear predictor is described using the following equation:

$$Y=\boldsymbol{X\beta}+\varepsilon$$

where $\varepsilon$ captures the difference between the linear predictor ($Y$) and the independent variable ($\boldsymbol{X\beta}$**)**.

Median regression (Median) has a similar equation as MLR, but depicts the relationship between the median of domain scores ($Q_{Y|X}\left( median \right)$) and the linear predictor. Censored regression models assume the boundaries of the dependent variable are due to censoring, i.e. the mean of latent PRO scores, denoted by $Y^{*}$ for Tobit, or the median of latent PRO scores, denoted by $Q_{Y^{*}|X}\left( median \right)$for CLAD, can exceed the upper and lower boundaries, but they are not observable. The CLAD is a subset of Median that estimates the median value of the parameters, whereas Tobit regression is an extension of MLR that estimates the mean. Both Tobit and CLAD describe the relationship between the latent variable and the linear predictor. The observed dependent variable of the censored regression is defined using the following equations:

$$Y=\left\{ \begin{aligned} a, &Y^{*}\leq a \\ Y^{*}, &a<Y^{*}<b \\ b, &b\leq Y^{*} \end{aligned} \right.$$

where $a$ and $b$ denotes the lower and upper bounds of the PRO score respectively.

Ordinal regression assumes the observed dependent variable ($Y^{*}$) is ordinal with $k$ possible ordered categories or levels $l:\{0, \ldots, k-1\}$, and the latent dependent variable ($Y$) is a continuous variable with a linear function of a series of independent variables ($\boldsymbol{X}$). Binomial regression is a generalisation from logistic regression, which assumes the conditional distribution of $Y_{i}$ of the $i$th subject given the number of success ($\theta_{i}$) obtained in $k$ binomial trials follows a binomial distribution, i.e. $Y_{i}\sim Bin(k,\theta_{i})$. The probability of success ($\theta_{i}$) is a random variable, which is assumed to follow a beta distribution for the beta-binomial regression (BB), and to follow a logit-Normal distribution for the binomial-logit-Normal regression (BLN). Fractional regression can be used to analyse bounded data on a continuous 0 to 1 scale. Under this category, we introduce the beta regression (BR) and fractional logistic regression (Frac), both of which assume a continuous ratio scattering between 0 and 1.

The inverse standard Normal cumulative distribution function, denoted by $\Phi^{-1}$, is used for the OP model:

$$g\left( \pi\right){=probit\left( \pi\right)=\Phi}^{-1}\left( \pi\right)=\boldsymbol{X\beta}$$

The logit link is used for the OL model, binomial regression and fractional regression in this study:

$$g\left( \pi\right)=logit\left( \pi\right)=ln(\frac{\pi}{1-\pi})=\boldsymbol{X\beta}$$

where $\pi$ is the distributional parameter in different methods.

Recoding of SF-36 domain scores to an ordinal form is required to run ordinal regression (OL and OP) and binomial regression (BB and BLN). The observed dependent variable of the ordinal regression and binomial regression is defined using the following equations:

$$Y=\left\{ \begin{aligned} 0, &0<Y^{*} \leq j_{1} \\ 1, &j_{1}< Y^{*} \leq j_{2} \\ \ldots, &{} \\ l_{k-2}, &j_{k-3}< Y^{*}\leq j_{k-2} \\ l_{k-1}, &j_{k-2}< Y^{*}\leq100 \end{aligned} \right.$$

where $j$ is a set of externally imposed endpoints of the observable categories, and $l$ is the recoded category score.

Similarly, recoding of the observed domain scores to a 0 to 1 scale is necessary to run fractional regression (BR and Frac). For Frac, the SF-36 scores on a $[a,b]$ scale need to be transformed to a closed interval [0, 1]. The equation to calculate the transformed score, denoted by $Y^{'}$, is shown below:

$$Y^{'}={(Y-a)}/{(b-a)}$$

As BR cannot account for scores at boundaries (i.e. a or b or 0 or 1), the SF-36 scores need to be ‘squeezed’ to an open interval (0, 1) using sample size, denoted by $N$, for adjustment. The transformed score, denoted by $Y^{''}$, is calculated using the following equation.

$$Y^{''}=[Y^{'}\left( N-1 \right)+0.5]/N$$

Appropriate recoding methods were applied for eight domain scores to accommodate the application of various statistical methods.

**Stata codes for recoding and regression analysis**

We provide the following codes to fit the ten statistical methods in our study, including MLR, Median, Tobit, CLAD, ordinal regression (OL and OP), binomial regression (BB and BLN), and fractional regression (Frac and BR). The SF-36 mental health (MH) score at 6-month follow-up from the Lifestyle Matters (LM) trial is used as an example for explanation.

**Variable Specification**

- mh6 is the score of SF-36 mental health score at 6-month follow-up in the LM trial.
- omh6 is the recoded ordinal score of mh6 to fit ordinal regression and the binomial regression;
- fmh6 is the recoded score of mh6 on a [0,1] scale to fit fractional logistic regression;
- bmh6 is the recoded score of mh6 on a (0,1) scale to fit beta regression;
- SZ is the sample size of patients;
- mh0 is the SF-36 mental health score at baseline;
- omh0 is the recoded ordinal score of mh0 to fit ordinal regression and the binomial regression;
- fmh0 is the recoded score of mh0 on a [0,1] scale to fit fractional logistic regression;
- bmh0 is the recoded score of mh0 on a (0,1) scale to fit beta regression;
- group is a binary variable for treatment (treatment group = 1, control group = 0);
- N is the number of possible observable values of a SF-36 domain score. In our example, N equals 21 for the SF-36 version 2 mental health domain.

**Stata codes**

** # Recoding methods

** To ordinal scale

generate omh0 = irecode(mh0, 2.5, 7.5, 12.5, 17.5, 22.5, 27.5, 32.5, 37.5, 42.5, 47.5, 52.5, 57.5, 62.5, 67.5, 72.5, 77.5, 82.5, 87.5, 92.5, 97.5)

generate omh6 = irecode(mh6, 2.5, 7.5, 12.5, 17.5, 22.5, 27.5, 32.5, 37.5, 42.5, 47.5, 52.5, 57.5, 62.5, 67.5, 72.5, 77.5, 82.5, 87.5, 92.5, 97.5)

** To [0,1] scale

gen fmh6 = mh6/100

gen fmh0 = mh0/100

** To (0,1) scale

egen SZ6 = count(mh6)

gen bmh6 = (fmh6*(SZ6-1)+0.5)/SZ6

egen SZ0 = count(mh0)

gen bmh0 = (fmh0*(SZ0-1)+0.5)/SZ0

**# regression analysis

** Multiple linear regression

regress mh6 group mh0

** Tobit regression

tobit mh6 group mh0, ll(0) ul(100)

** Censored least absolute deviation regression [package ‘sg153’ is required]

clad mh6 group mh0, rep(1000) ul(100)

** Median regression

qreg mh6 group mh0

** Ordered logit model

ologit omh6 group omh0

** Ordered probit model

oprobit omh6 group omh0

** Beta-binomial regression [package ‘betabin’ is required]

betabin omh6 group omh0, n(N) link(logit)

** Binomial-logit-Normal regression

glm omh6 group omh0, link(logit) family(binomial N)

** Fractional logistic regression

fracreg logit fmh6 group pmh0

** Beta regression

betareg bmh6 group bmh0

**Post estimation plots for multiple linear regression and Tobit regression**

A series of post estimation plots were generated to test model assumptions where possible, including homoscedasticity and Normality of residuals for post-estimation of multiple linear regression (MLR) and Tobit regression, shown in Figure S1-S4.

**Figure S1 Residual plot against fitted values after MLR estimation of SF-36 domain scores**

1. **COPD**


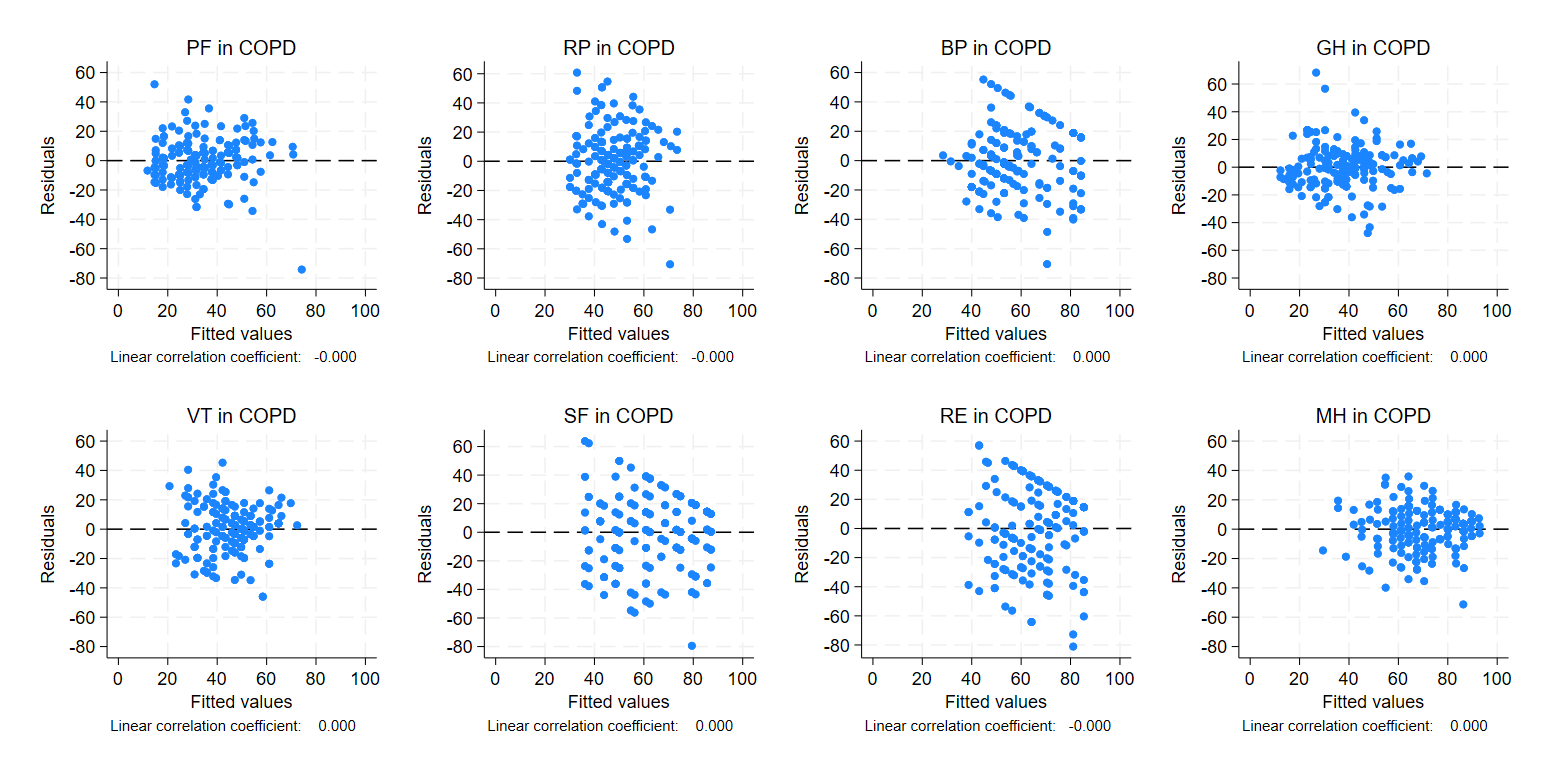


*Continue on the next page*

**Figure S1 Residual plot against fitted values after MLR estimation of SF-36 domain scores (continued)**

1. **LM**


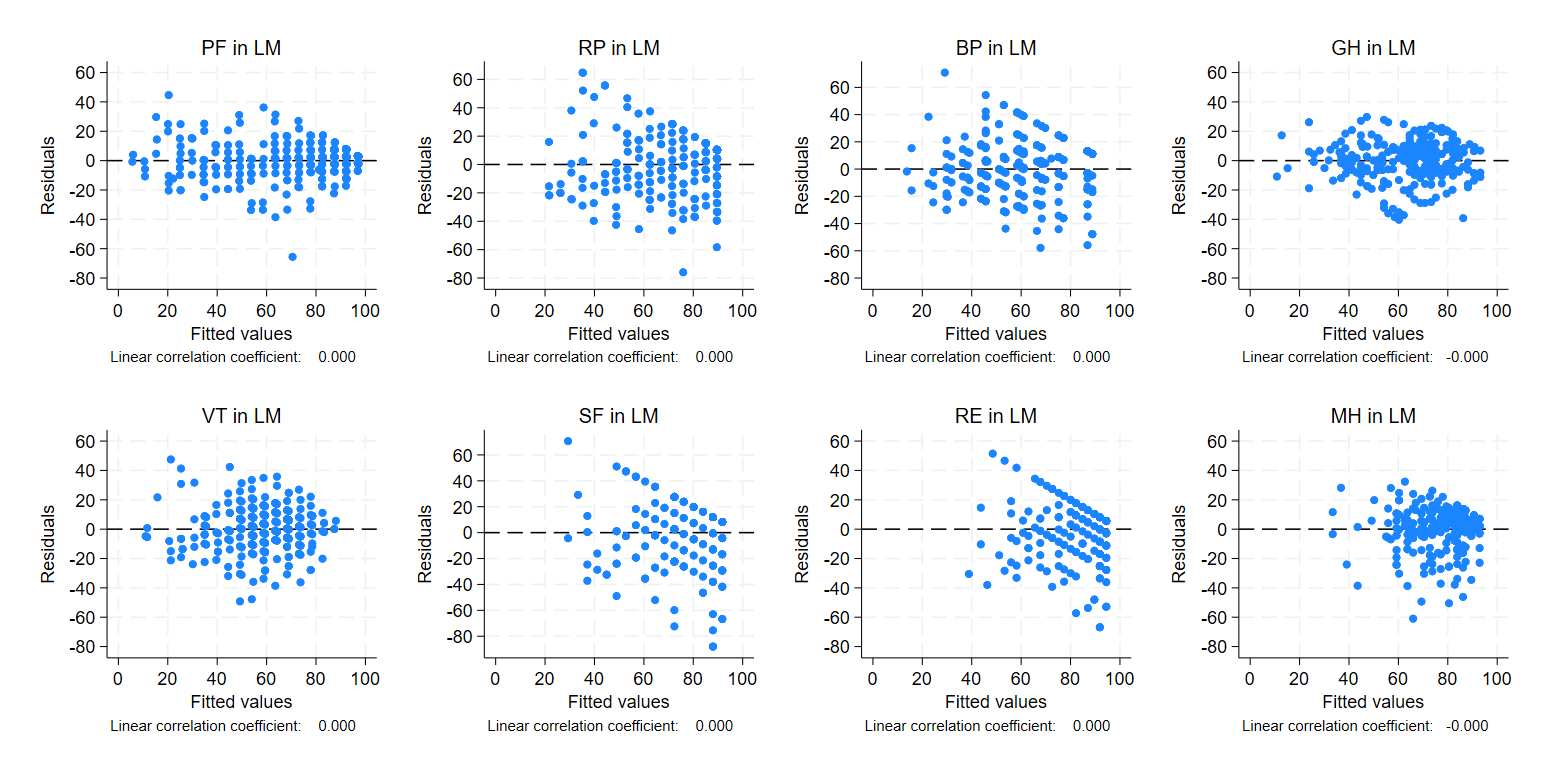
*Continue on the next page*

**Figure S1 Residual plot against fitted values after MLR estimation of SF-36 domain scores (continued)**

1. **PLINY**


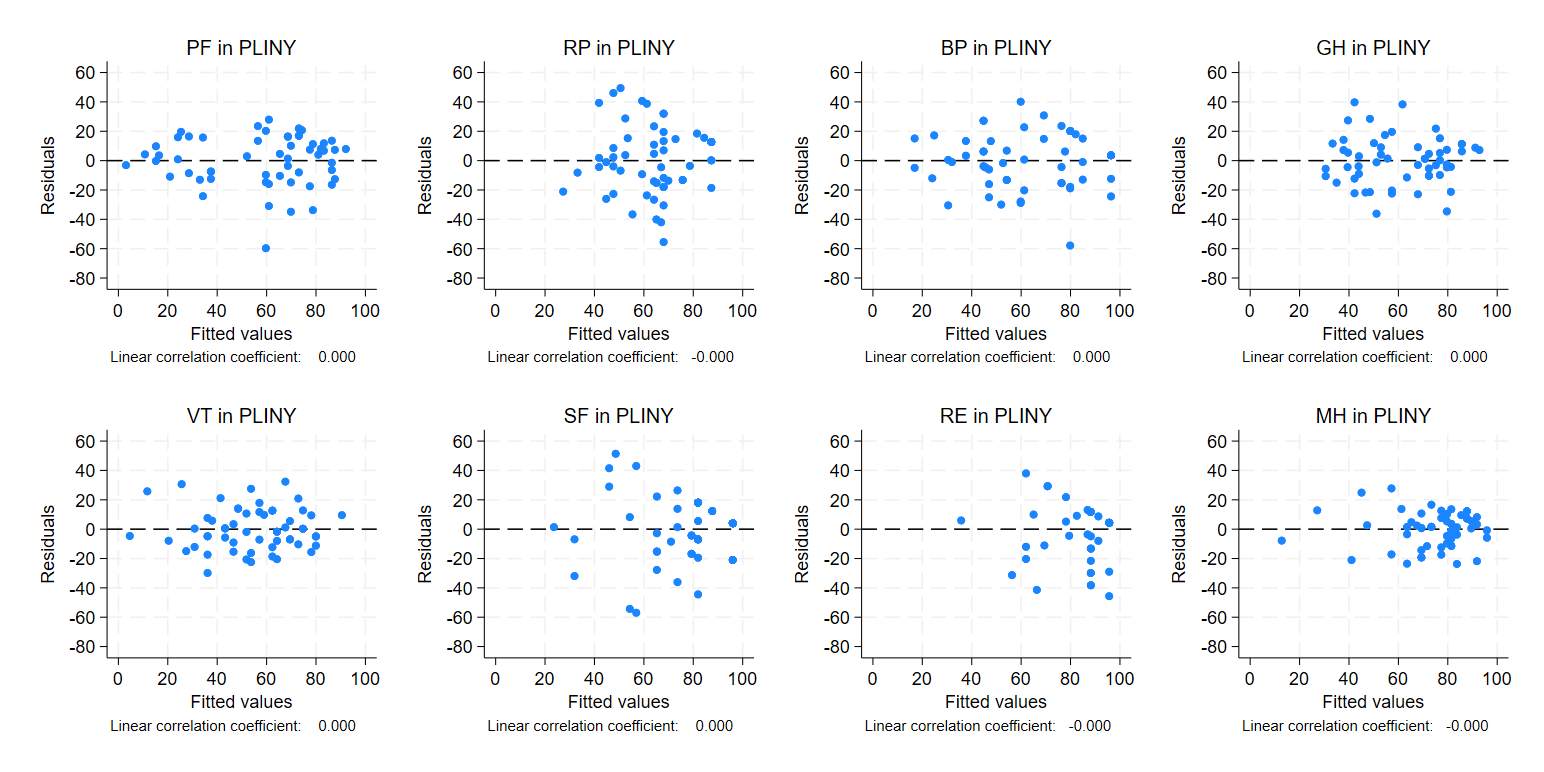


Horizontal lines represent residuals equal zero. BP, bodily pain; GH, general health; MH, mental health; MLR, multiple linear regression; PF, physical functioning; RE, role limitation – emotional; RP, role limitation – physical; SF, social functioning; SF-36, Short Form-36; VT, vitality.

**Figure S2 Histogram of residuals after MLR estimation of SF-36 domain scores**

1. **COPD**


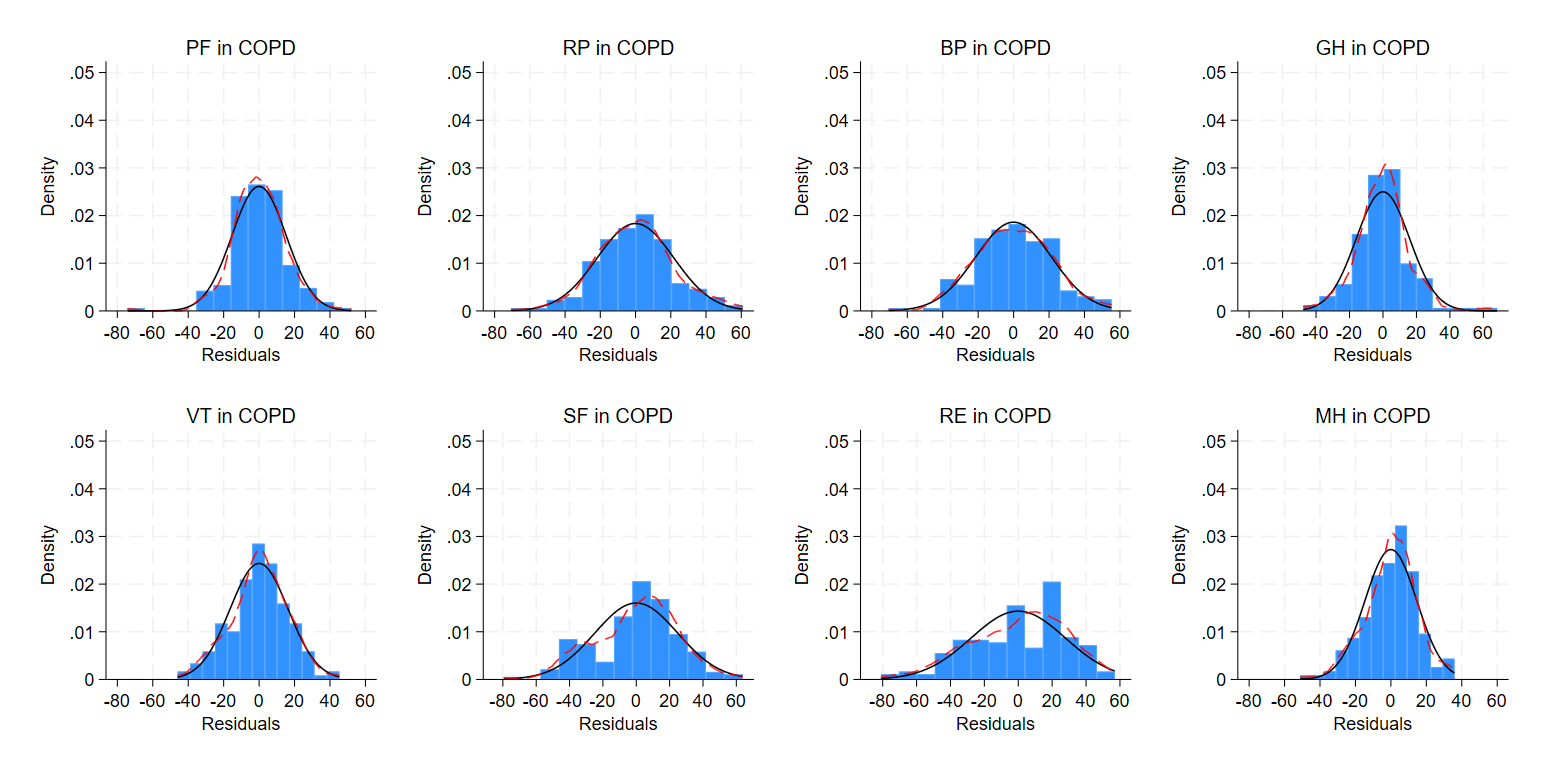


*Continue on the next page*

**Figure S2 Histogram of residuals after MLR estimation of SF-36 domain scores (continued)**

1. **LM**


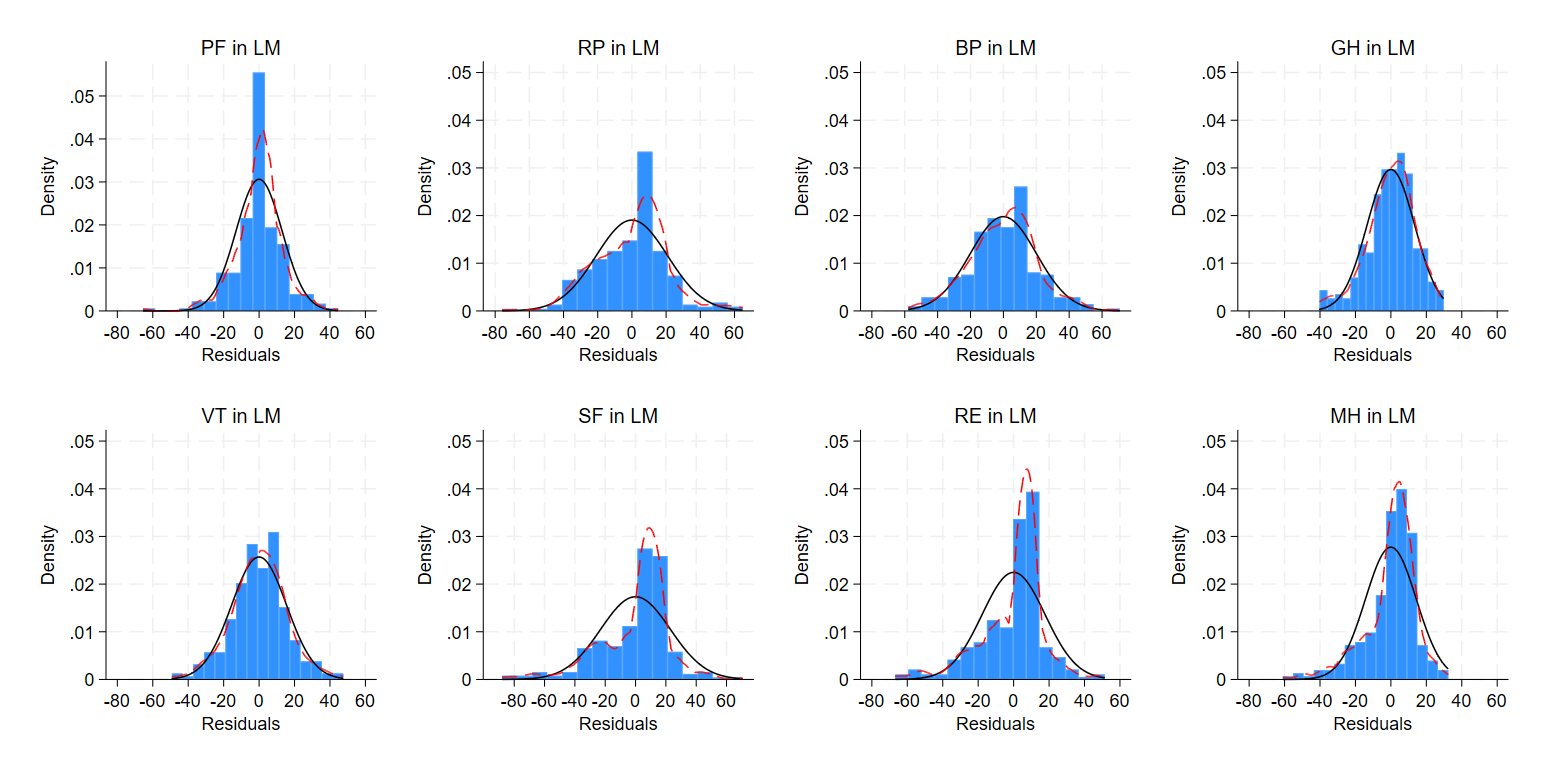


*Continue on the next page*

**Figure S2 Histogram of residuals after MLR estimation of SF-36 domain scores (continued)**

1. **PLINY**


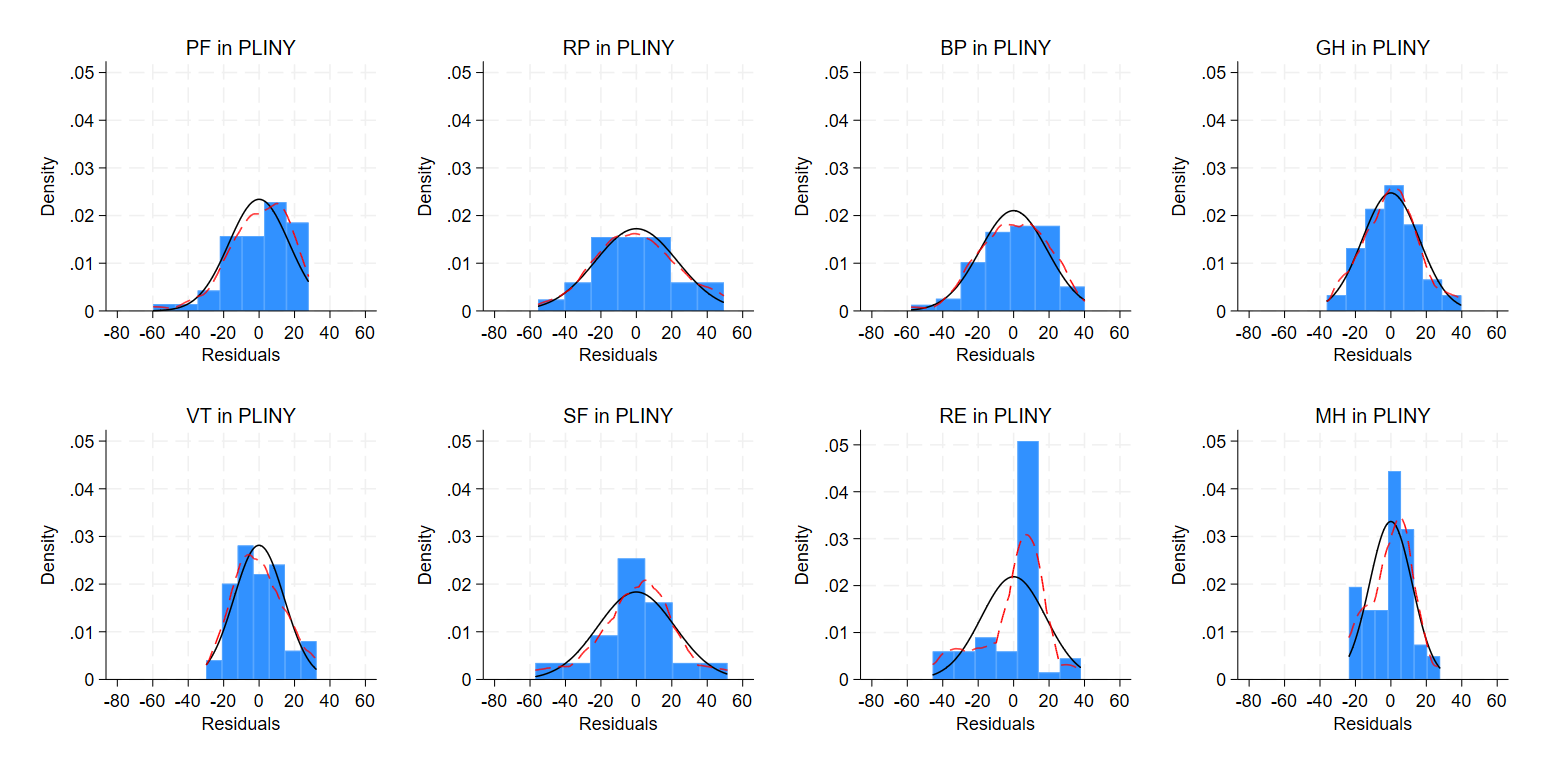


Red dashed curves depict the kernel density estimate of the distribution, while the black curves represent a normal distribution. BP, bodily pain; GH, general health; MH, mental health; MLR, multiple linear regression; PF, physical functioning; RE, role limitation – emotional; RP, role limitation – physical; SF, social functioning; SF-36, Short Form-36; VT, vitality.

**Figure S3 Residual plot against fitted values after Tobit estimation of SF-36 eight domain scores**

1. **COPD**


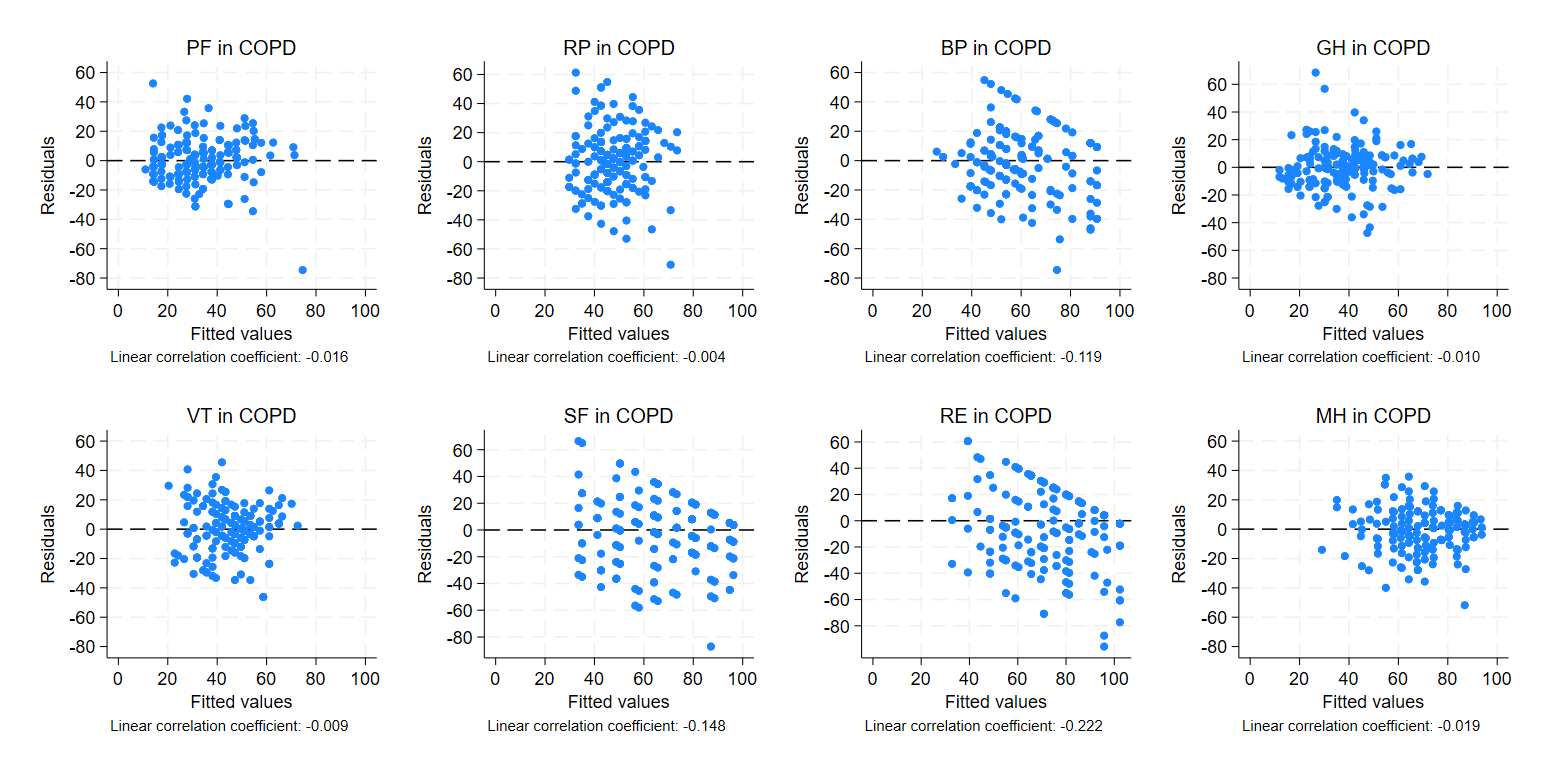
*Continue on the next page*

**Figure S3 Residual plot against fitted values after Tobit estimation of SF-36 eight domain scores (continued)**

1. **LM**


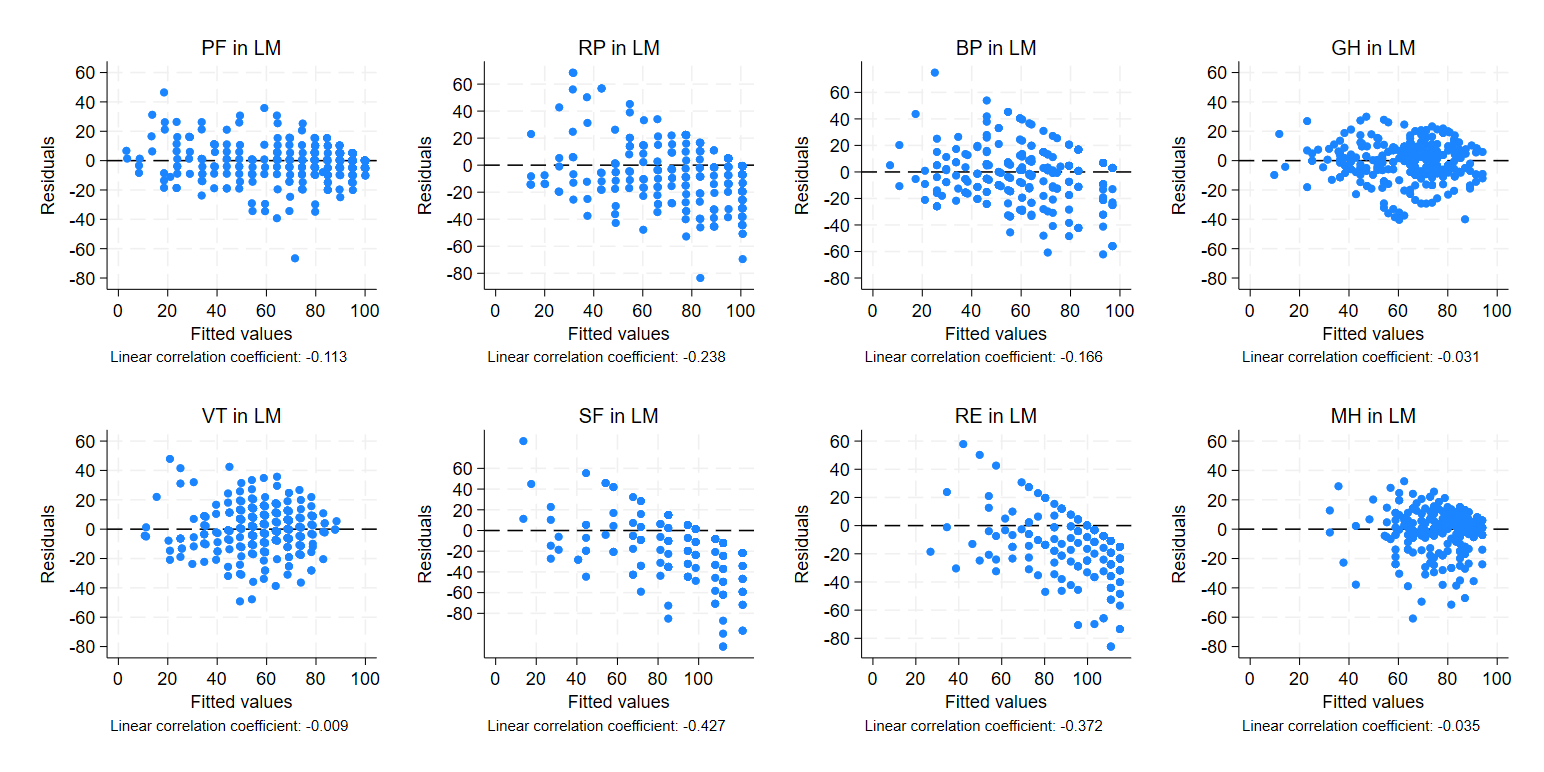


*Continue on the next page*

**Figure S3 Residual plot against fitted values after Tobit estimation of SF-36 eight domain scores (continued)**

1. **PLINY**


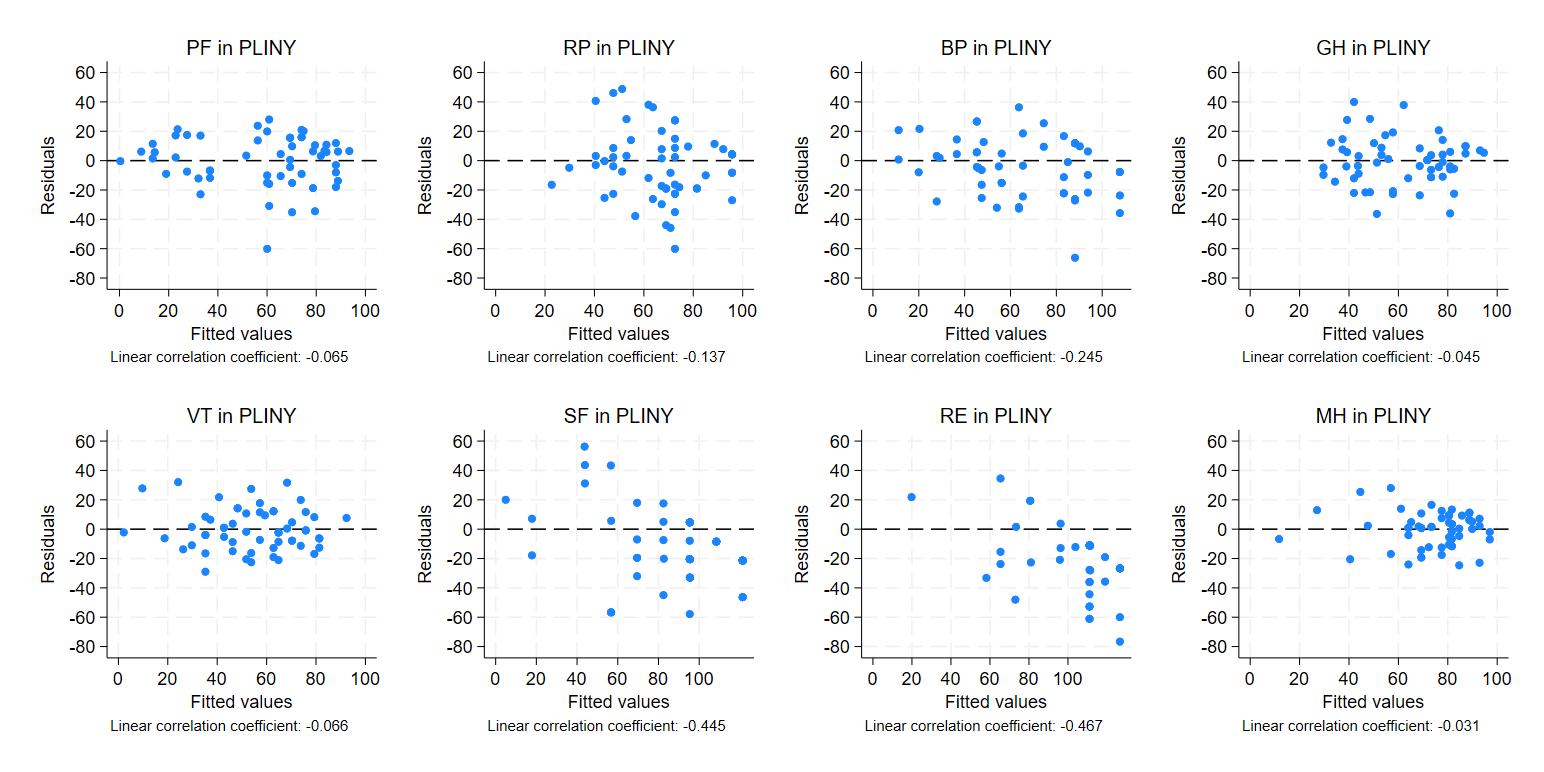


Horizontal lines represent residuals equal zero. BP, bodily pain; GH, general health; MH, mental health; PF, physical functioning; RE, role limitation – emotional; RP, role limitation – physical; SF, social functioning; SF-36, Short Form-36; Tobit, Tobit regression; VT, vitality.

**Figure S4 Histogram of residuals after Tobit estimation of SF-36 eight domain scores**

1. **COPD**


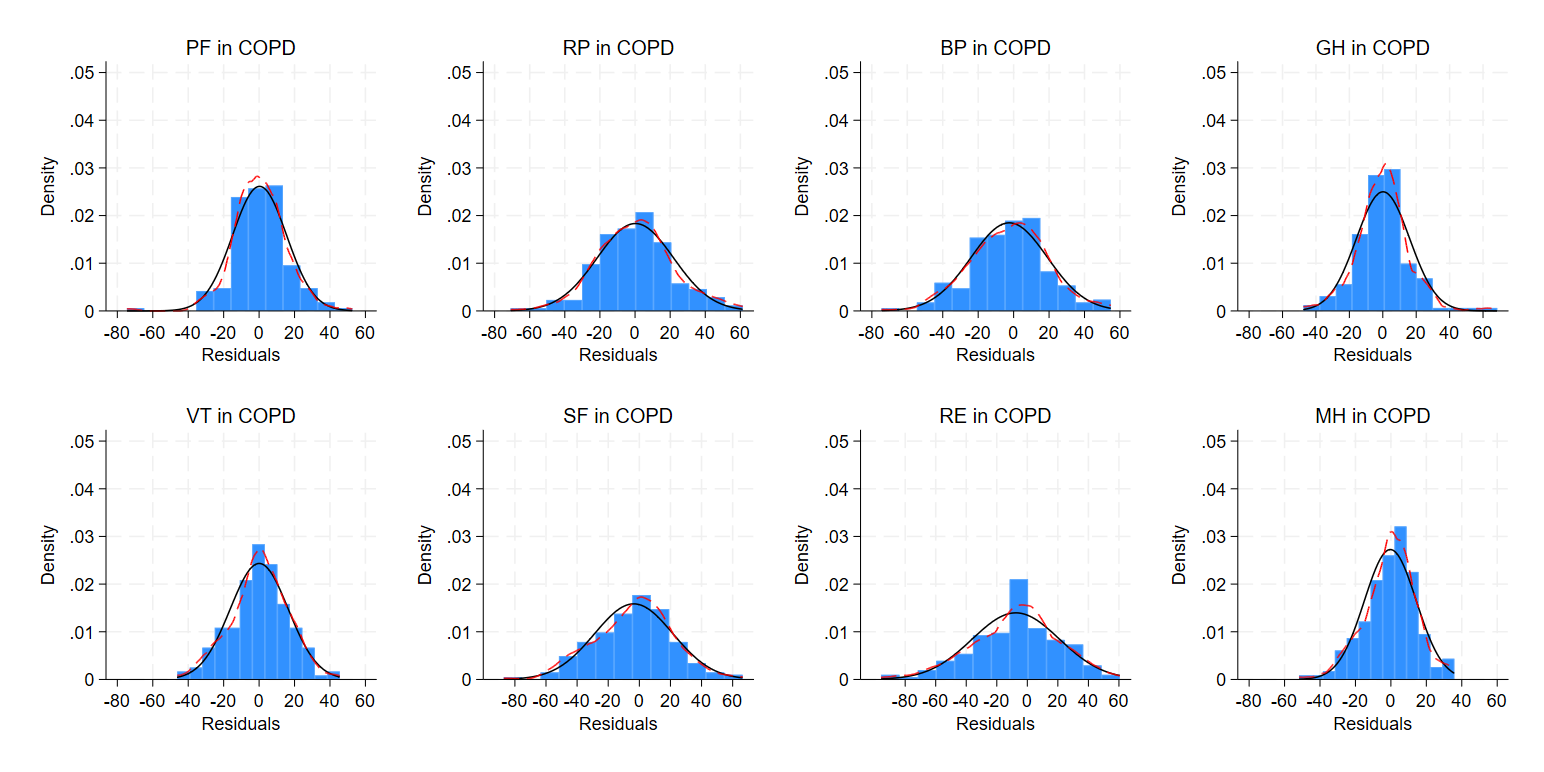


*Continue on the next page*

**Figure S4 Histogram of residuals after Tobit estimation of SF-36 eight domain scores (continued)**

1. **LM**


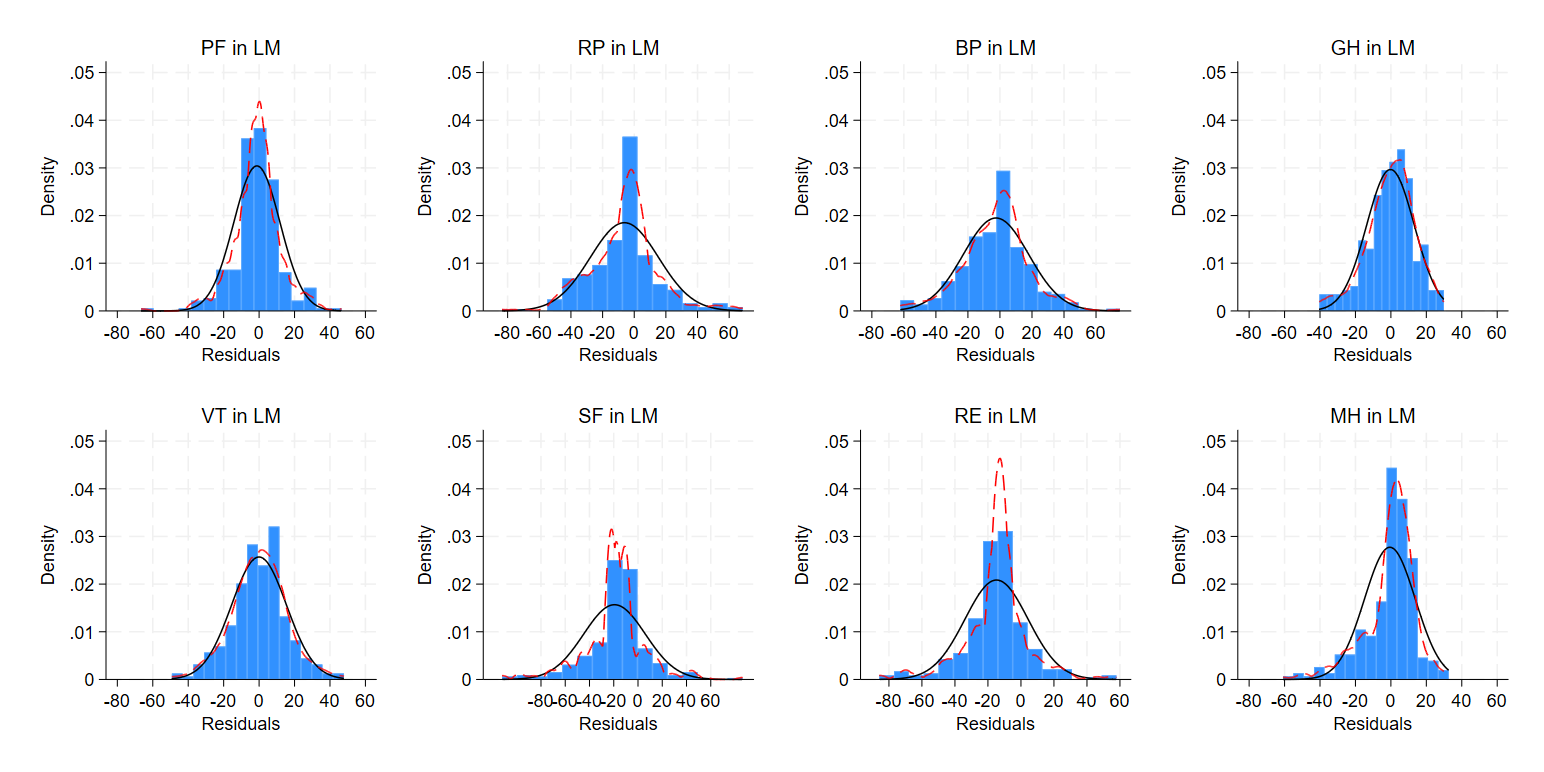


*Continue on the next page*

**Figure S4 Histogram of residuals after Tobit estimation of SF-36 eight domain scores (continued)**

1. **PLINY**


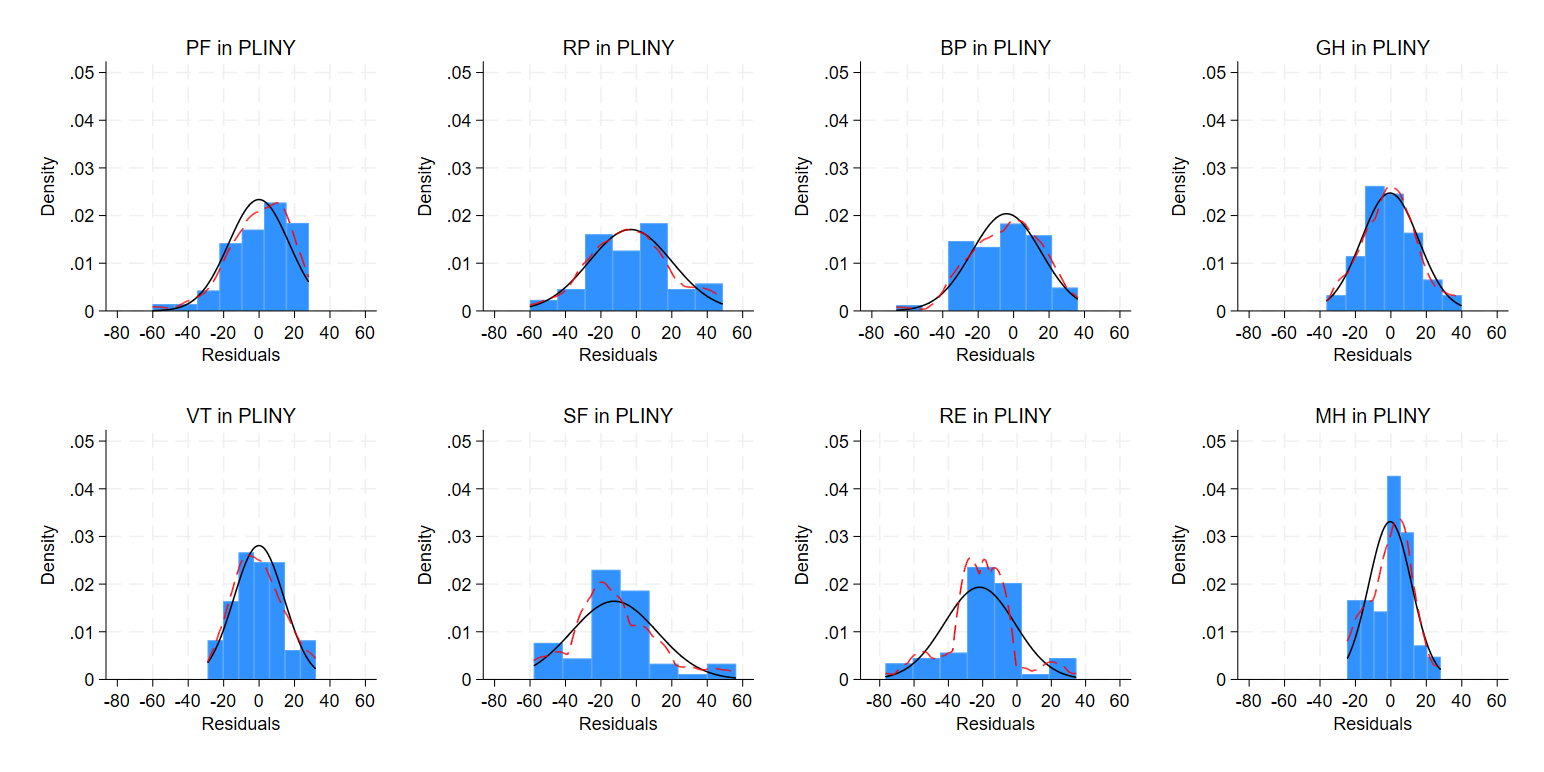


Red dashed curves depict the kernel density estimate of the distribution, while the black curves represent a normal distribution. BP, bodily pain; GH, general health; MH, mental health; PF, physical functioning; RE, role limitation – emotional; RP, role limitation – physical; SF, social functioning; SF-36, Short Form-36; Tobit, Tobit regression; VT, vitality.

**Effect size plots for SES of eight domains in three RCTs**

Effect size plots are produced for standardised effect size with its associated 95% confidence intervals (CIs) of treatment estimates by ten different statistical methods for SF-36 domain scores using as the secondary outcome in included trials, shown in Figure S5.

**Figure S5 SES with 95% CIs of treatment estimates for SF-36 domain scores**

1. **COPD**

*Continue on the next page*

**Figure S5 SES with 95% CIs of treatment estimates for SF-36 domain scores (continued)**

1. **LM**

*Continue on the next page*

**Figure S5 SES with 95% CIs of treatment estimates for SF-36 domain scores (continued)**

1. **PLINY**

The bars on two sides of the vertical line for each method represents the 95% confidence intervals (CIs) for SES, which are calculated using $SES\pm1.96\times SE(SES)$, where $SE\left( SES \right)$ represents the standard error of the SES. The red horizontal line represents the SES for no different between two treatment arms (i.e. y = 0). BB, beta-binomial regression; BLN, binomial-logit-normal regression; BP, bodily pain; BR, beta regression; CLAD, censored absolute least deviation regression; Frac, fractional logistic regression; GH, general health; OP, ordered probit model; OL, ordered logit model; PF, physical functioning; RE, role limitation – emotional; RP, role limitation – physical; Median, median regression; MH, mental health; MLR, multiple linear regression; Tobit, Tobit regression; SF, social functioning; VT, vitality.
